# Pro-Arrhythmic Potential of Oral Antihistamines (H1): Combining Adverse Event Reports with Drug Utilization Data across Europe

**DOI:** 10.1371/journal.pone.0119551

**Published:** 2015-03-18

**Authors:** Elisabetta Poluzzi, Emanuel Raschi, Brian Godman, Ariola Koci, Ugo Moretti, Marija Kalaba, Bjorn Wettermark, Miriam Sturkenboom, Fabrizio De Ponti

**Affiliations:** 1 Department of Medical and Surgical Sciences, Alma Mater Studiorum—University of Bologna, Bologna, Italy; 2 Division of Clinical Pharmacology, Karolinska Institutet, Stockholm, Sweden; 3 Strathclyde Institute of Pharmacy and Biomedical Sciences, Strathclyde University, Glasgow, United Kingdom; 4 Clinical Pharmacology Unit, University of Verona, Verona, Italy; 5 Republic Fund for Health Insurance, Belgrade, Serbia; 6 Centre for Pharmacoepidemiology, Karolinska University Hospital, Solna, Stockholm, Sweden; 7 Stockholm, County Council, Stockholm, Sweden; 8 Erasmus University Medical Centre, Rotterdam, Netherlands; University of Minnesota, UNITED STATES

## Abstract

**Background:**

There is appreciable utilisation of antihistamines (H_1_) in European countries, either prescribed by physician and purchased by patients for self-medication. Terfenadine and astemizole underwent regulatory restrictions in ’90 because of their cardiac toxicity, but only scarce clinical data are available on other antihistamines.

**Aim:**

To investigate the pro-arrhythmic potential of antihistamines by combining safety reports of the FDA Adverse Event Reporting System (FAERS) with drug utilization data from 13 European Countries.

**Methods:**

We identified signals of antihistamine arrhythmogenic potential by analyzing FAERS database for all cases of Torsades de Pointes (TdP), QT abnormalities (QTabn), ventricular arrhythmia (VA) and sudden cardiac death/cardiac arrest (SCD/CA). Number of cases ≥3 and disproportionality were used to define alert signals: TdP and QTabn identified stronger signals, whereas SCD/CA identified weaker signals. Drug utilization data from 2005 to 2010 were collected from administrative databases through health authorities and insurance.

**Results:**

Antihistamines were reported in 109 cases of TdP/QT prolongation, 278 VA and 610 SCD/CA. Five agents resulted in stronger signals (cetirizine, desloratadine, diphenhydramine, fexofenadine, loratadine) and 6 in weaker signals (alimemazine, carbinoxamine, cyclizine, cyproeptadine, dexchlorpheniramine and doxylamine). Exposure to antihistamines with stronger signal was markedly different across European countries and was at least 40% in each Country. Cetirizine was >29 Defined Daily Doses per 1000 inhabitants per day (DID) in Norway, desloratadine >11 DID in France and loratadine >9 DID in Sweden and Croatia. Drugs with weaker signals accounted for no more than 10% (in Sweden) and in most European countries their use was negligible.

**Conclusions:**

Some second-generation antihistamines are associated with signal of torsadogenicity and largely used in most European countries. Although confirmation by analytical studies is required, regulators and clinicians should consider risk-minimisation activities. Also antihistamines without signal but with peculiar use in a few Countries (e.g., levocetirizine) or with increasing consumption (e.g., rupatadine) deserve careful surveillance.

## Introduction

There is appreciable utilisation of antihistamines in European countries principally for the treatment of allergies. Their main therapeutic effects are mediated by their activity on H_1_ receptors in immunoregulating cells, CNS, and smooth muscle. [1].

Their pharmacological profile can be grouped into either first-generation antihistamines, e.g., diphenhydramine, and second-generation, e.g., cetirizine and loratadine. First-generation agents readily reach the CNS, have high affinity for central H_1_ receptors and are mainly used in the treatment of disorders related to the vomiting centre (motion sickness, post-operative or drug-induced nausea and vomiting, vertigo, etc.) and for sedation (insomnia and anaesthesia). Second-generation antihistamines only partially cross the blood brain barrier and are preferred in allergic disorders (urticaria, conjunctivitis, rhinitis and hay fever) because of the lack of central side effects [2]. Many administration routes are available for prescribing or self purchasing. Pharmacological properties, indications, route of administration and formulations strongly influence the safety of use of antihistamines.

Drowsiness is the most frequent consequence of the oldest agents, whereas second-generation antihistamines were developed to minimise this effect [1]: only high doses or predisposing factors can impair patients’alertness. Antagonism of muscarinic, serotoninergic and alpha-adrenergic transmission are responsible for other central and peripheral effects including, for instance, blurred vision, urinary retention, constipation, weight gain, and orthostatic hypotension. These side-effects are also more frequent for the first- rather than for the second-generation agents.

Cardiac toxicity is less frequent than the side-effects described above. However, it is potentially more severe for patients, with no well defined differences between first and second-generation antihistamines.

Because of the relatively low incidence, the arrhythmogenic risk has principally been evaluated in preclinical models rather than in patients. In fact, astemizole and terfenadine represented probably the first examples of widely used drugs withdrawn or strongly restricted in the label due to risk of QT prolongation. This regulatory measure was also based on the contemporary marketing approval of many second-generation agents perceived as safer for cardiac risk (e.g., fexofenadine, represents the active metabolite of terfenadine, and was especially developed to avoid the interaction with cardiac potassium channels and relevant ventricular arrhythmia). The accurate clinical evaluation on the arrhythmogenic potential has become mandatory before marketing authorisation (i.e., Thorough QT study—TQT) since 2005. So far, though, these studies have only been conducted for three agents, namely bilastine, levocetirizine and rupatadine, and in all cases provided negative results [3–6].

Almost all other antihistamines have not been included among drugs with this risk (absence from Arizona CERT list, crediblemeds.org). The only exception is diphenhydramine, which is included in the third list (i.e., conditional risk of TdP: substantial evidence supports the conclusion that these drugs prolong QT and have a risk of developing TdP, but only under certain known conditions).

Summary of Product Characteristics of medicinal products containing antihistamines do not highlight this possible risk, apart from the inclusion of “tachycardia” among rare adverse events in the side effect paragraph. No mention of proarrhythmic risk in cautions was found.

## Aim

To primarily investigate the pro-arrhythmic potential of antihistamines in practice, by combining the analysis of safety reports of the FDA Adverse Event Reporting System (FAERS) with drug utilization data from 13 European Countries.

The analysis of spontaneous reporting data will identify antihistamines with alert signals for arrhythmia. Drug utilization data among European Countries will estimate population exposure to these drugs in order to map the level of pro-arrhythmic potential due to antihistamine utilization. The secondary aim is to update physicians and regulators about possible differences in the proarrhythmic potential of antihistamines.

## Methods

### Ethics Statement

All data analyses performed in this retrospective study are based on anonymized data, which did not allow the identification of individual patients. The study dealt with two independent data sources, namely (a) spontaneous reports, which are publicly available from the FAERS database and (b) drug utilization data, which can be accessed by other researchers only upon request to relevant personnel. Therefore, submission to and approval by the Institutional Review Board was waived.

### Pharmacovigilance Data

The FDA Adverse Event Reporting System (FAERS) database contains over 4 million reports of Adverse Drug Reactions (ADRs) of worldwide human drugs and biological products and offers public access to raw data starting from 2004. By virtue of its large population coverage (including all US reports and serious/unexpected reports from European Countries) and free availability, FAERS is an attracting source to explore rare adverse drug reactions (such as arrhythmias) and highly informative of the global pattern of arrhythmogenic events. [7–10].

In a recent study [9], we described the consensus process to define the arrhythmogenic potential of drugs within the FP7 funded ARITMO project (www.aritmo-project.org). In line with this approach, we decided to investigate the arrhythmogenic potential of antihistamines by collecting all cases reported in the 2004–2011 period on: torsades de pointes (TdP), QT abnormalities (both symptomatic and asymptomatic), ventricular arrhythmia and sudden cardiac death/cardiac arrest (respectively VA and SCD/CA). In particular, we first analyzed cases of TdP or QT abnormalities (TdP/QTabn) as they are strongly intertwined with a high degree of drug-attributable risk, whereas we considered separately ventricular arrhythmia and cardiac arrest/sudden cardiac death (as they are not necessarily correlated with each other, have a lower degree of drug-attributable risk, but represent possible symptomatic consequences of severe QT prolongation and can increase the sensitivity of the analysis).

The Reporting Odds Ratio (ROR) with 95% CI was calculated for each group of events; disproportion was considered in case of 95% Confidence Interval (95CI) (one-tailed) lower limit >1. In addition, concomitant drugs were taken into account in order to better characterize each single case. We checked for co-reporting of cardiovascular classes (digitalis: C01A; Class I/III antiarrhythmics: C01B; diuretics: C03; beta blockers: C07; calcium channel blockers: C08 and ACE inhibitors/ARBs: C09) and lists I and II of the AZCERT program (crediblemeds.org). AZCERT program currently represents the most authoritative source of evidence on the torsadogenic risk of drugs and it was, therefore, used as a reference to establish the notoriety of the arrhythmogenic risk, in order to discuss the novelty of the signals.

In order to allow conjunction analyses with drug utilization data and provide a public health perspective, antihistamines were classified based on pharmacovigilance data as follows.

- Stronger signals: at least 3 cases without concomitant proarrhythmic drugs and ROR lower limit >1for TdP/QTabn.
- Weaker signals: at least 3 cases without concomitant proarrhythmic drugs (see above) and ROR lower limit >1for VA or CA/SCD.

### Drug Utilization Data

After identification of pharmacovigilance signals from FAERS, drug utilization data allowed us to map the risk derived from exposure to antihistamines in European Countries [10]. In this study, data were collected from administrative databases through health authorities and health insurance personnel across Europe. Consistent and reliable data were obtained from 13 European Countries, which allowed adequate estimation of European population exposure since the 13 Countries have differences in geography and financing of healthcare: i.e. comprised Western (Austria, France, Italy, Norway, Spain [Catalonia], Sweden, and the UK [England and Scotland]), as well as Central and Eastern (Croatia, Serbia, Slovenia, Estonia, Lithuania) European Countries and regions. With only a few exceptions, administrative databases contained data on reimbursed prescriptions in ambulatory care and covered the entire population (see also Raschi et al. [10] for details of data characteristics in each specific Country).

Total dispensed data (ATC code: R06) were expressed as defined daily doses (DDD) per 1,000 inhabitants per day (now referred to as DID) for the 2005–2009/2010 period. In the analyses, we considered a detectable use of at least 0.01 DID. The mean annual DID value was used to estimate the actual population exposure over the period of interest and to distinguish antihistamine use on the basis of pharmacovigilance signals. A time-trend analysis was also carried out, where appropriate.

## Results

### Pharmacovigilance Data

Overall, 27 antihistamines were reported in at least one case of the outcomes of interest. They were found in 109 cases of TdP/QT abnormalities (the most specific case definition); loratadine, cetirizine and diphenhydramine accounted for the highest number of cases (26) and in particular loratadine and cetirizine were reported in 17 and 13 cases of TdP, respectively (Table 1). Eight drugs resulted in a ROR significantly >1 for these outcomes, but only 5 were reported in at least 3 cases without concomitant confounding factors/drugs: cetirizine, desloratadine, diphenhydramine, fexofenadine, loratadine (Table 1). On the basis of our signal definition, these five drugs present stronger alert signals.

**Table 1:** Disproportionality analysis for antihistamines with cases of QT prolongation/TdP.

	Cases of TdP; QTs; QTa	*ROR*	*CI lower*	*CI upper*	*Cases without concomitant AZCERT drugs or CV drugs*
alimemazine	0; 2; 3	9.14	3.75	22.28	2
azelastine	0; 1, 0	*na*	*na*	*na*	1
cetirizine	13; 4; 9	3.36	2.28	4.96	18
desloratadine	0; 2, 5	3.20	1.52	6.74	5
dexchlorpheniramine	5; 5; 2	7.00	3.94	12.41	2
diphenhydramine	6; 4; 16	1.99	1.35	2.92	12
doxylamine	0; 0; 1	na	na	na	1
ebastine	0; 1, 0	na	na	na	1
epinastine	1; 0; 0	na	na	na	0
fexofenadine	2; 2; 8	4.03	2.28	7.13	5
ketotifen	0; 0; 4	2.87	1.07	7.69	2
levocetirizine	0; 0; 3	*2*.*63*	*0*.*84*	*8*.*19*	3
loratadine	17; 4; 5	4.79	3.25	7.07	8
meclozine	1; 0; 0	*na*	*na*	*na*	0
mepyramine	0; 0; 1	*na*	*na*	*na*	1
promethazine	2; 2; 5		*0*.*91*	*3*.*39*	1
rupatadine	1; 0; 4	347.30	93.24	1293.68	0

NOTES: in italic, not statistically significant ROR; na = number of cases <3; TdP = Torsades de Pointes, QTs = symptomatic QT prolongation, QTa = asymptomatic QT prolongation.

By considering also more sensitive outcomes, antihistamines were reported in 278 cases of VA (Table 2) with the 60% represented by non-fatal ventricular tachycardia (non-fatVT), whereas cases of CA/SCD were 610, covered for the 90% by CA reports (Table 3). Diphenhydramine was largely the most reported agent in both outcomes, followed by cetirizine and loratadine in VA series and loratadine, doxylamine, dexchlorfeniramine and fexofenadine in CA/SCD list.

**Table 2:** Disproportionality analysis for antihistamines with cases of ventricular arrhythmia.

	cases of VF; fatVT; non-fatVT	*ROR*	*CI lower*	*CI upper*	*cases without concomitant AZCERT or CV drugs*
alimemazine	0;4;0	*1*.*83*	*0*.*68*	*4*.*95*	1
azelastine	1;0;2	*1*.*48*	*0*.*47*	*4*.*65*	2
brompheniramine	0;1;1	*0*.*82*	*0*.*20*	*3*.*30*	1
buclizine	0;0;1	*na*	*na*	*na*	0
carbinoxamine	0;1;2	*2*.*47*	*0*.*78*	*7*.*83*	3
cetirizine	7;3;24	*1*.*11*	*0*.*79*	*1*.*56*	20
chlorphenamine	0;0;5	*1*.*00*	*0*.*41*	*2*.*41*	5
cyclizine	1;0;8	*22*.*54*	*10*.*63*	*47*.*77*	4
desloratadine	1;1;26	*3*.*35*	*2*.*29*	*4*.*89*	24
dexbrompheniramine	0;1;1	*14*.*02*	*3*.*07*	*63*.*99*	2
dexchlorpheniramine	0;13;13	*3*.*94*	*2*.*65*	*5*.*85*	19
diphenhydramine	6;33;53	*1*.*81*	*1*.*47*	*2*.*22*	50
doxylamine	0;3;1	*1*.*04*	*0*.*39*	*2*.*80*	2
ebastine	0;0;1	*na*	*na*	*na*	1
epinastine	1;0;1	*1*.*77*	*0*.*44*	*7*.*22*	2
fexofenadine	1;3;19	*1*.*97*	*1*.*30*	*2*.*99*	15
ketotifen	0;0;1	*na*	*na*	*na*	1
levocetirizine	0;1;4	*1*.*11*	*0*.*46*	*2*.*69*	4
loratadine	5;3;24	1.49	1.05	2.12	26
meclozine	0;0;1	*na*	*na*	*na*	0
pheniramine	1;0;0	*na*	*na*	*na*	0
thiethylperazine	0;1;1	*1*.*89*	*0*.*26*	*13*.*81*	2
triprolidine	0;0;1	*na*	*na*	*na*	1

NOTES: in italic, not statistically significant ROR; na = number of cases <3;VF = ventricular fibrillation; fatVT = fatal or life-threatening ventricular fibrillation; non-fatVT = other VT cases.

**Table 3:** Disproportionality analysis for antihistamines with cases of cardiac arrest (CA) or sudden cardiac death (SCD).

	cases of CA; SCD	*ROR*	*CI lower*	*CI upper*	*cases without concomitant AZCERT or CV drugs*
alimemazine	7;1	2.77	1.36	5.63	4
azelastine	4;1	*1*.*84*	*0*.*75*	*4*.*49*	4
brompheniramine	3;3	*1*.*85*	*0*.*82*	*4*.*18*	6
carbinoxamine	8;4	8.13	4.42	14.95	12
cetirizine	14;6	*0*.*48*	*0*.*31*	*0*.*74*	15
chlorcyclizine	0;1	na	na	na	0
chlorphenamine	3;4	*1*.*03*	*0*.*49*	*2*.*18*	6
cyproheptadine	15;0	8.49	4.92	14.66	1
desloratadine	10;3	*1*.*11*	*0*.*64*	*1*.*93*	11
dexchlorpheniramine	27;8	3.97	2.82	5.60	21
dimetindene	0;1	*na*	*na*	*na*	0
diphenhydramine	366;21	6.13	5.52	6.82	215
doxylamine	34;2	7.83	5.51	11.12	18
ebastine	2;0	*2*.*78*	*0*.*67*	*11*.*55*	2
fexofenadine	24;10	2.17	1.54	3.06	9
levocetirizine	5;1	*0*.*98*	*0*.*44*	*2*.*21*	6
loratadine	39;4	1.48	1.09	2.01	17
meclozine	3;0	*1*.*13*	*0*.*36*	*3*.*54*	2
thiethylperazine	0;1	*na*	*na*	*na*	1

NOTES: in italic, not statistically significant ROR; na = number of cases <3

Almost all signals emerging from analysis of TdP/QT abnormalities were confirmed by both groups of more sensitive outcomes (i.e., VA and SCD). The following additional drugs showed signal for at least one outcome group: cyclizine and dexchlorpheniramine appeared as a signal by considering the cases of VA, whereas alimemazine, carbinoxamine, cyproeptadine and doxylamine appeared with the outcomes of CA or SCD (Table 4). These latter 6 drugs could be included in a list of weaker signals.

**Table 4:** Integration of results from different outcomes for signal identification.

	At least 3 Cases of TdP; QTs; QTa	*Significant ROR of TdP-QT*	*At least* 3 Cases of VA	*Significant ROR of VA*	*At least* 3 Cases of CA or SCD	*Significant ROR of* CA or SCD	*Labeled AZCERT*	*Signal*
alimemazine	Y^1^	Y	Y	N	Y	Y		Weaker
azelastine	N	N	Y	N	Y	N		
brompheniramine	N	na	N	na	Y	N		
buclizine	na	na	N	na	Na	na		
carbinoxamine	na	na	Y	N	Y	Y		Weaker
cetirizine	Y	Y	Y	N	Y	N		Stronger
chlorphenamine	Na	na	Y	N	Y	N		
cyclizine	Na	na	Y	Y	Na	na		Weaker
cyproheptadine	Na	na	na	na	Y	Y		Weaker
desloratadine	Y	Y	Y	Y	Y	N		Stronger
dexbrompheniramine	Na	na	N	na	Na	na		
dexchlorpheniramine	Y^1^	Y	Y	Y	Y	Y		Weaker^2^
diphenhydramine	Y	Y	Y	Y	Y	Y	III	Stronger
doxylamine	N	na	Y	N	Y	Y		Weaker
ebastine	N	na	N	na	N	na		
epinastine	N	na	N	na	Na	na		
fexofenadine	Y	Y	Y	Y	Y	Y		Stronger
ketotifen	Y^1^	Y	N	na	Na	na		*borderline* ^2^
levocetirizine	Y	N	Y	N	Y	N		
loratadine	Y	Y	Y	Y	Y	Y		Stronger
meclozine	N	na	N	na	Y	N		
mepyramine	N	na	na	na	Na	na		
pheniramine	Na	na	N	na	Na	na		
promethazine	Y^1^	N	na	na	Na	na		
rupatadine	Y^1^	Y	na	na	Na	na		*borderline* ^2^
thiethylperazine	na	na	N	na	N	na		
triprolidine	na	na	N	na	Na	na		

^*1*^ *less than 3 cases without AZCERT drugs or CV drugs*; QTs = symptomatic QT prolongation, QTa = asymptomatic QT prolongation.

^2^ stronger signal was not reached only due to lack of 3 cases without *AZCERT drugs or CV drugs*

### Drug Utilization Data

**Fig 1:**
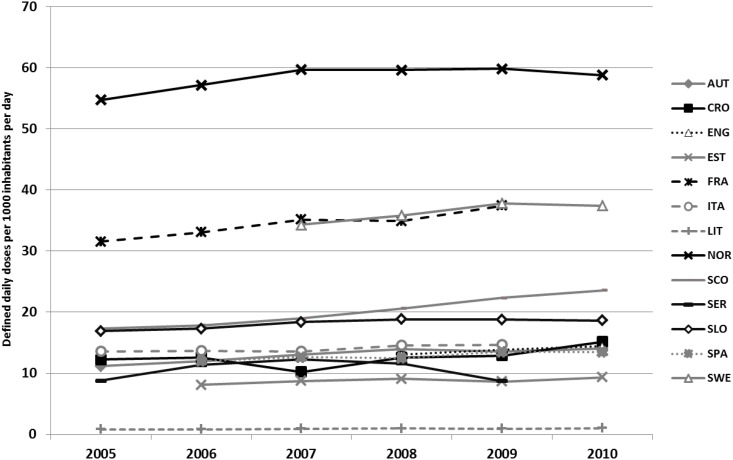
Cross-National Comparison: time trend. AUT: Austria; CRO: Croatia; EST: Estonia; FRA: France; IT: Italy; LIT: Lithuania; NOR: Norway; SPA: Spain (Catalonia); SCO: Scotland; SER: Serbia; SLO: Slovenia; SWE: Sweden. ENG: 2008–2010; EST: 2006–2010; FRA: 2005–2008; ITA: 2006–2010; SPA: 2006–2010; SWE: 2007–2010. Data from Norway and Sweden include also hospital data. Data from Sweden also includes OTC data.

Considerable heterogeneity was observed in antihistamine utilization across Europe: e.g., in 2009, this ranged from 0.9 DID in Lithuania to 8.7 in Estonia, 18.8 in Slovenia, 22.4 in Scotland, 37.4 in France and 59.9 in Norway (Fig. 1). However, there was an appreciable increase in antihistamine utilisation throughout the study period. This ranged from a 7% increase in Norway to 21% in Austria (2010 vs 2005). Serbia is an exception, with a peak in 2007 but a negligible variation throughout the study period. Concerning the contribution of different supplying regimens to the collected data, Serbia and Sweden showed very different situations. In Serbia, total utilization including dispensed prescriptions of drugs not covered by the Health Insurance Fund was appreciably higher than reimbursed utilisation (8.8 to 12.3 total vs. 2.0 to 3.2 reimbursed utilisation), whereas in Sweden, there was lower over the counter (OTC) use than reimbursed utilisation (10.1 to 11.6 OTC and 23.3 to 24.6 reimbursed).

**Fig 2:**
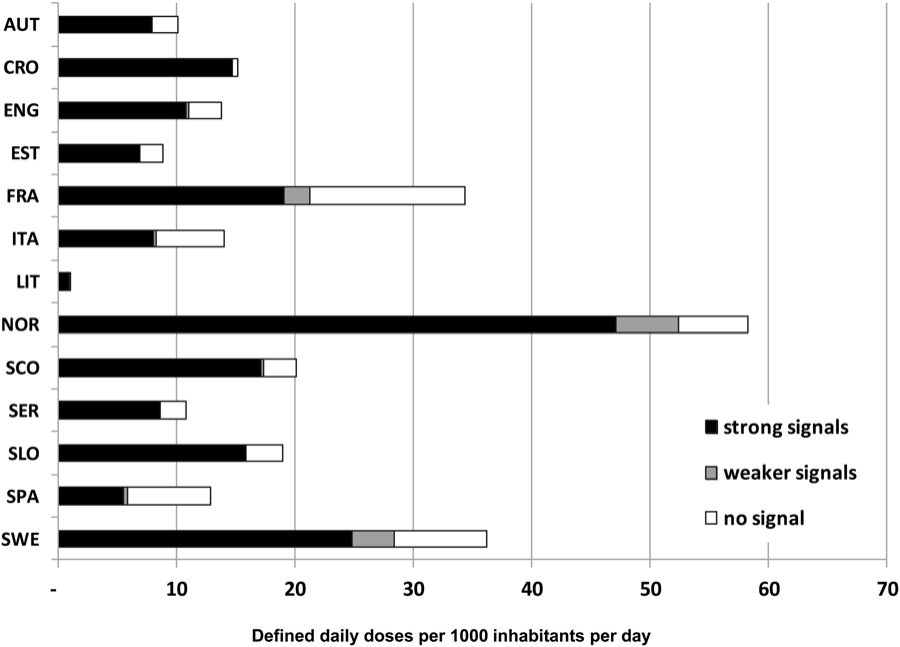
Antihistamine utilisation on the basis of pharmacovigilance signals.

**Fig 3:**
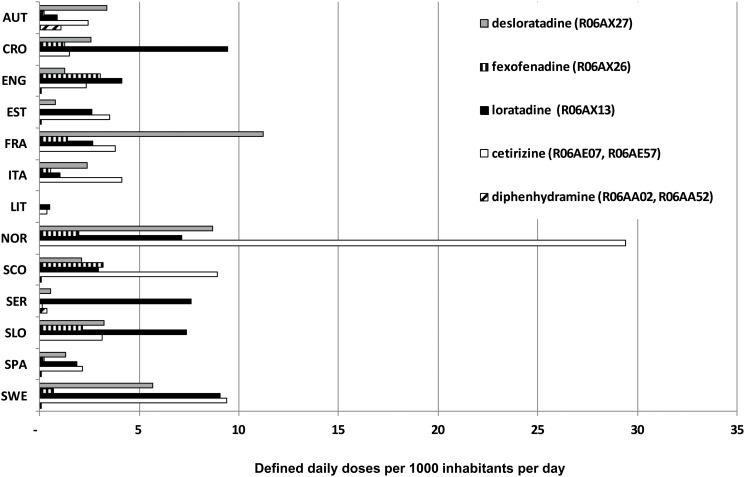
Utilisation of different antihistamines with stronger signals.

Antihistamines with stronger signals represented a very different percentage of the total antihistamine utilization in each Country, e.g. ranging from 43% in Spain (Catalonia) to 97% in Croatia (Fig. 2). Among these, cetirizine was >29 DID in Norway, desloratadine >11 DID in France and loratadine >9 DID in Sweden and Croatia (Fig. 3). Drugs with weak signals accounted for no more than 10% (in Sweden) and in most Countries their use was negligible.

## Discussion

This study attempts, for the first time, to combine analysis of drug utilisation and pharmacovigilance data to interpret signals of arrhythmogenicity related to antihistamines. Our findings showed signals of arrhythmogenicity especially for second-generation agents for which no previous pharmacovigilance data were published: cetirizine, desloratadine, fexofenadine, loratadine. Among old agents, only diphenhydramine fulfilled criteria for signal generation. These five drugs covered more than 40% of total consumption of antihistamines in each European Country, ranging from 43% in Spain to 97% in Croatia. On the other hand, utilisation of drugs with weaker signal (cyclizine, dexchlorpheniramine, alimemazine, carbinoxamine, cyproeptadine and doxylamine) was negligible, except for Sweden, Norway and France where it ranged from 6 to 10% of total antihistamine utilisation.

Concerning pharmacovigilance results, it should be reminded that generated signals represent input to systematically review all available case-reports in the literature [11] and to perform formal analytical studies (i.e., case-control studies) to confirm or refuse actual causal-effect relationship in order to better substantiate possible regulatory and clinical decisions; on the other hand, signals can *per se* stimulate attention of regulators and in some cases justify regulatory interventions before confirmation of safety concerns, especially when therapeutic alternatives with solid evidence of safety are available on the market. In this context, Countries with overall high use of antihistamines with unexpected signals (i.e., Croatia, Norway, England and Sweden) should probably take measures to limit population exposure to these drugs. Also the contribution to total exposure from OTC use should be taken into account by regulators, because of difficult patient monitoring by physicians. As a matter of fact, differences seen between Serbia and Sweden may be due to differences in patient co-payments (high vs. low OTC consumption).

When findings on antihistamines are compared with those in other therapeutic areas, some general issues should be first considered: the absolute number of cases of arrhythmia is low (especially against antipsychotics and antibiotics [10;12]); patients exposed to antihistamines have probably less risk factors for arrhythmia (most prevalent indications for antihistamine use are mild and not related to cardiovascular comorbidities); on the other hand, strict monitoring of patients treated with antihistamines is rare and this may contribute to the lack of identification of non-severe events (e.g., asymptomatic QT abnormalities). Therefore, reports of arrhythmia by antihistamines are more likely to have a higher drug-attributable component: antihistamines induced arrhythmia less frequently than other drugs, probably because exposed patients had no additional risk factors rather because intrinsically lower risk.

Reasons listed above can potentially explain the lack of antihistamines in the AZCERT list. In fact, only diphenhydramine is included in the III list (i.e., weak evidence of risk, which may be clinically relevant only in susceptible patients). All other antihistamines, therefore, generated unknown signals and each of them should be specifically discussed. With regard to desloratadine, a recent prescription event monitoring (PEM) [13] showed no evidence for arrhythmia when used in general practice in England. On fexofenadine, the case of TdP reported in 1999 by Pinto et al. [14] remained the only one in the literature attributed to this agent and all articles published later excluded a drug-attributable risk of arrhythmia [15–17]. On the other hand, it should be acknowledged that fexofenadine monotherapy (i.e. without any concomitant drug) was reported in a not negligible percentage of cases of arrhythmia recorded in FDA Adverse Event Reporting System: 5 out of 12 cases of TdP/QTabn, 15 out of 24 VA cases and 7 out of 34 of CA/SCD cases. As for loratadine and cetirizine, some published papers reported cases of arrhythmia in patients taking poly-pharmacy or overdose [18–22], but the specific contribution of antihistamines in the adverse events was not finally identified. The debate is still ongoing, but so far large observational analyses [23] and preclinical studies [24–26] failed to demonstrate a role of these two agents in cardiac repolarisation impairments.

In the risk—benefit assessment of each medicine, also indication and setting of use should be taken into account. In particular, some antihistamines are used especially as antinausea/antiemesis drugs, e.g., promethazine and cyclizine. For these drugs, the peculiar clinical setting, i.e., palliative care in patients with cancer or critically-ill patients, may facilitate the occurrence of TdP and a comparison with alternative drugs used in the same conditions, e.g., ondansetron, could better clarify their possible proarrhythmic risk.

Some antihistamines, although not showing clear signals in current analysis, ask for continuing surveillance. Agents largely used only in a few countries also need further investigation on their proarrhythmic risk in the national database. In particular, the very high use of levocetirizine in France (53% of the total defined daily doses of antihistamines) and the fact that most FAERS cases of arrhythmia reporting levocetirizine are from France (8/14) suggests the need to specifically analyse the French spontaneous report databases-. Differences in safety profile, and in signal generation, between enantiomer and racemic mixture (i.e., levocetirizine and cetirizine) should not surprise because of possible differences in doses, metabolism and stereoselective targets [27].

Surveillance is also recommended for antihistamines, which met some of the considered criteria, but did not reach the full definition of signal. This is especially the case of rupatadine, which was marketed in 2008 and shows steadily increasing use: e.g. Spain and Italy showed a consumption of 0.71 and 0.38 defined daily doses in the observed time-window. On the basis of our data, rupatadine was reported in 1 case of TdP and 4 of asymptomatic QT prolongation, reporting odds ratio resulted statistically significant, but in most cases known proarrhythmic drugs were co-prescribed. Information of its proarrhythmic profile includes also a Thorough QT study [6], which provided negative results, and, by contrast, a recent warning from the Italian Medicine Agency on some spontaneous reports of cardiac rhythm impairment [http://www.agenziafarmaco.gov.it/sites/default/files/Rupatadina_29.5.2013.pdf].

High consumption of antihistamines in Norway and Sweden found in our study is in line with a previous drug utilisation survey [28], which proposed a higher data quality as potential reason for this finding. In this regard, each Country should check for data quality before definitively deciding regulatory strategies (e.g., Lithuania should verify its very low consumption of antihistamines, which may be due to issues of reimbursement restrictions similar to the situation for proton pump inhibitors and statins versus Western European countries [29]).

## Strengths and Limitations

Intrinsic limitations of pharmacovigilance analyses are well known and should be briefly acknowledged here: [11] while modern adverse event reporting into FAERS suggests that so-called Weber effect and stimulated reporting do not significantly and systematically affect spontaneous reporting analyses [30;31], the issue of under-reporting together with the lack of data on population exposure actually do not allow calculation of incidence rate of ADRs from spontaneous reporting systems, even when they are as large as FDA Adverse Event Reporting System. Therefore, signal detection asks *per se* for confirmation through additional analytical studies or systematic review of clinical data with relevant meta-analyses, especially for rare events. Our specific methodology attempted to characterize statistically significant signals (i.e., disproportionality formally obtained) considering additional qualitative information (i.e., number of cases without confounders) and the clinical relevance of the event of interest (i.e., TdP, which carries stronger drug-related component as compared to ventricular arrhythmia) in order to prioritize signals for potential regulatory consideration (e.g., according to the extent of local drug use).

Also drug utilisation data suffer from well-known limitations: discrepancy between supplied and actually administered drugs is common to all drug utilisation data collections. Moreover, in some countries, only reimbursed prescriptions can be collected, and reimbursement systems are heterogeneous across Europe. Also when Countries are able to collect sales data, hospital data could be missing, although for some drug classes (e.g., antihistamines) they represent only a low percentage of the total consumption. Despite limitations, the DDD system is a recognized tool for standardizing doses in drug utilization research and is currently recommended by the WHO for international drug utilization studies [32].

Although also serious and rare European reports are submitted to the FDA, our data may be affected by specific drug marketing penetration (e.g., drugs only marketed in Europe). In addition, the characterization of signals is dynamic so that a given antihistamine may change strength of signal (i.e., strong vs weak) depending on the availability of new pharmacovigilance data over time. This is particularly important for drugs classified as weak or borderline signals, which therefore require monitoring in the next years to fully appreciate their actual post-marketing safety profile.

This study attempted to overcome the lack of information on exposed population in spontaneous reporting sources, by linking FAERS findings with drug consumption. It should be kept in mind that results derived from a combination of both data sources must be interpreted very cautiously, because information are obtained from separate databases, which are affected by different bias (see above). In addition, the link between drug utilization data and spontaneous reports is not straightforward. In most of the cases, adverse events track the magnitude of utilization, meaning that the volume of spontaneous reports largely mirror the prescribing trend. As a matter of fact, it should be acknowledged that antihistamines with strong signal are also largely and consistently used across Europe.

However, the aim of our study was to approach pharmacovigilance data (derived by the clinical pharmacology perspective of single patients) in a population risk perspective and to provide risk weighting at the population level. Although pharmacovigilance and drug utilization data sources did not cover the same geographical area (only Europe for drug utilization and theoretically all Countries for pharmacovigilance), we used these two dataset for different purposes: (a) FAERS, the largest public pharmacovigilance database, to accurately classify and characterize torsadogenic signals by antihistamines, and (b) European drug utilization data to map the overall antihistamine use (i.e., estimate the European population exposure).

## Conclusions

Combined analysis of pharmacovigilance and drug utilisation data can provide useful elements for clinicians and regulators in terms of population perspectives of safety concerns of drugs.

Some antihistamines resulted in signals of torsadogenic risk and most of them are largely used especially in some European Countries.

National Agencies should focus their attention on own peculiar uses of antihistamines and define strategies to minimise proarrhythmic potential, also in the light of their multiple place in therapy and the difficulty in monitoring their use by physicians because of the high frequency of self-medication. Educational initiatives focussed on recognition of patient susceptibility and possible differences in the potential risk among single agents should be addressed.
